# Sexual violence as a predictor of unintended pregnancy among married young women: evidence from the 2016 Nepal demographic and health survey

**DOI:** 10.1186/s12884-019-2342-3

**Published:** 2019-06-07

**Authors:** Kiran Acharya, Yuba Raj Paudel, Pramita Silwal

**Affiliations:** 1 New ERA, Rudramati Marga, Kalopul, Kathmandu, 44621 Nepal; 2 School of Public Health, University of Alberta, Edmonton, Canada; 3 Nepal Red Cross Society, Kalimati, Kathmandu, 44600 Nepal

**Keywords:** Sexual violence, Unintended pregnancy, Young women, Nepal

## Abstract

**Background:**

Sexual violence in marital relationship is higher among women married at young age. Although sexual violence has been found to increase risk for unintended pregnancy, there is a limited published data from Nepal linking sexual violence with unintended pregnancy. The current study aimed to investigate association of partner sexual violence with unintended pregnancy among young married women who experienced child birth in last 5 years.

**Methods:**

Using data from Nepal Demographic and Health Survey, we studied the prevalence of sexual violence and unintended pregnancy, and their association among 560 married women (weighted sample) of 15–24 years who gave childbirth in last 5 years of the survey. We used multivariate logistic regression to analyse the association of sexual violence and other factors with unintended pregnancy. Analysis was conducted considering inverse probability weighting, clustering, and stratification to provide unbiased estimates of the population parameters.

**Results:**

Nearly a quarter of women (22.7%) reported to have experienced unintended pregnancy in the last 5 years of the survey and almost one in 10 women (9%) reported to have ever experienced sexual violence from their husbands. Women who ever experienced sexual violence from their husbands were at 2.3 times higher odds to report an unintended pregnancy (aOR = 2.3; 95% CI = 1.1–4.8) compared to women who did not experience sexual violence from their husbands independent of important socio-demographic variables and ever use of contraception.

**Conclusion:**

The strong association of sexual violence within marital relationship with unintended pregnancy among young women in Nepal necessitates the provision of comprehensive sexual and reproductive health services. Women need routine assessment, and referral to appropriate services for sexual violence to reduce unintended pregnancy and its consequences.

## Background

Sexual violence is widespread and deceptive problem that has serious physical, psychological, emotional and social consequences [1]. WHO defined sexual violence broadly as: “any sexual act, attempt to obtain a sexual act, unwanted sexual comments or advances, or acts to traffic, or otherwise directed, against a person’s sexuality using coercion, by any person regardless of their relationship to the victim, in any setting, including but not limited to home and work” [2]. Sexual violence against women does not only violate the rights of women and girls, but also limits their participation in society, and damages their health and well-being.

Previous studies have shown a relationship of domestic violence with abortion and unintended pregnancy [3–5]. A complex and multidirectional relationship is suggested to exist between induced abortion and violence [3]. A study from 4 Indian states (Bihar, Jharkhand, Maharastra, and Tamil Nadu) found that physical violence was associated with higher risk of induced abortion; and induced abortion was found to be leading to sexual and verbal violence [3]. A further analysis of DHS data found that women who experienced intimate partner violence (IPV) were more than twice the risk to abort their fetus in Azerbaijan and Moldova, whereas such women in Ukraine were nearly 5 times more likely to continue to unwanted live birth than women who did not ever experience physical and sexual violence from their husbands [5]. Similarly, secondary analysis of DHS data from Pakistan also found a higher likelihood of pregnancy loss(still birth, abortion) and unwanted pregnancy among women who experienced either emotional violence or physical violence or both [4]. Additionally, further analysis of DHS data from 10 countries found that women with history of intimate partner violence were 1.48–1.75 times more likely to terminate pregnancy than women not experiencing IPV [6].

Nearly half of the women in South Asia face violence in their home [7]. Violence is an inevitable reality of women’s lives since social and cultural norms that support violence have been institutionalized at all levels of their society: family, community, and state [7]. Violence against women is associated with underlying social, cultural, religious and gender norms and with the political instability [8]. One in two young married women reported to have experienced sexual violence within marriage in Nepal [9] and sexual violence is more common phenomenon than physical and emotional violence [10]. Adolescent and young women were more likely to have experienced sexual violence during early phase of their married life [11]. Research has documented negative consequences of sexual violence on physical and psychological health outcomes among Nepalese women [9].

Despite being a common phenomenon in Nepalese society, limited evidence is available on the association between sexual violence and unintended pregnancy in Nepal. It was estimated that 230,000–342,000 unintended pregnancies occurred in Nepal in 2011 [12]. Both the cross-sectional and prospective studies have revealed adverse consequences of unintended pregnancy to maternal and child health [12, 13]. Previous studies on correlates of unintended pregnancy in Nepal do not explore the association of sexual violence with pregnancy intendedness [14–16]. In this article we report on the prevalence of sexual violence from husbands in Nepal, and its association with unintended childbirth among currently married young women of 15–24 years who experienced child birth in last 5 years of the 2016 NDHS survey. We studied this age group because girls married at adolescent age were more likely to experience physical and sexual violence compared to those who were married at adult age [11, 17]. The findings will help policy makers to make informed decision for reducing the burden of unintended pregnancy in Nepal.

## Methods

We analyzed data from the 2016 Nepal Demographic and Health Survey (NDHS) which is a nationally representative survey. Women in the reproductive age group (15–49 years) from 11,040 households were interviewed in the 2016 NDHS survey. The survey involved the use of a three-stage stratified sampling technique. Stratification was achieved by separating each province into urban and rural areas.

### Sample selection

**Fig. 1:**
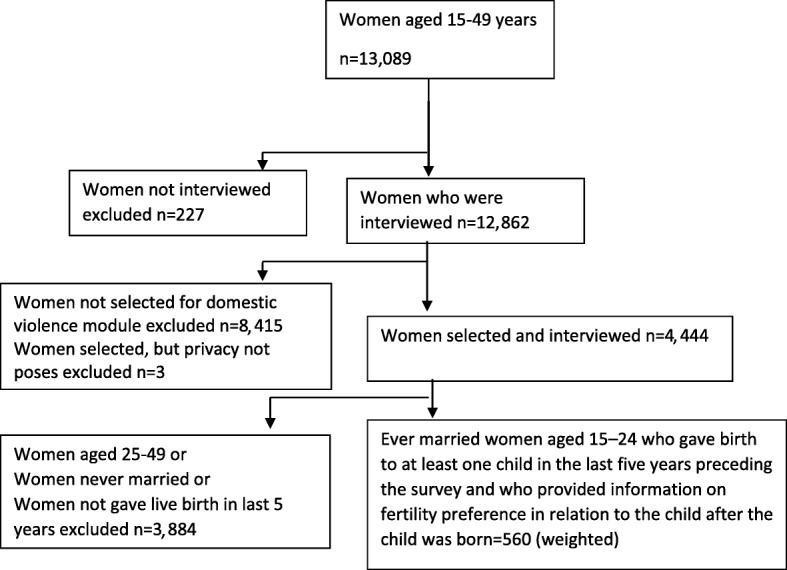
Flow chart showing selection of study sample

From the sample households, women from selected households were chosen for administering domestic violence module. The unit of analysis for this study were women aged 15–24 (*n* = 560 weighted cases) who gave birth to at least one child in the last 5 years preceding the survey and who provided information on fertility preference in relation to the child after the child was born (Fig.1). The domestic violence module was randomly administered to only one woman in a household if there were more than one woman in a household.

## Definition of variables

### Outcome variable

Precise measurement of birth intentions was essential to define fertility behavior of women. Measurement of pregnancy intention in the DHS was based on a question to women for every live birth: ‘When you were pregnant with [Name of the child] whether you wanted the pregnancy then, later or not at all?’. Women responding their last birth was ‘wanted later’ or ‘not wanted at all’ were characterized to have an unintended pregnancy and those who responded ‘wanted birth then’ were considered to have an intended pregnancy. Unintended pregnancy was coded ‘1’ and intended pregnancy was coded ‘0’. The terms ‘unintended pregnancy’ and ‘unintended birth’ are interchangeably used in this paper.

### Independent variables

Eligible women were asked 3 questions: “Did you ever experience physical force by husbands to have sexual intercourse when you did not want to?”, “Did your husband use physical force to perform any other sexual acts when you did not want to?”; and “Were you ever forced by your husband with threats or in any other way to perform sexual acts when you did not want to?”. Ever experience of sexual violence (yes, no) was derived from the response to above 3 questions. If a women answered ‘yes’ to at least one of the above 3 questions, then it was considered as having experienced sexual violence. If a women answered ‘No’ to all 3 questions, then it was considered as no experience of sexual violence from husbands. Ever experience of sexual violence was coded ‘1’ for ‘yes’ and ‘0’ for ‘no’.

Other covariates including socio-demographic characteristics, and reproductive health behavior of respondents and husband’s characteristics used in the current analysis were drawn from the literature [18–21]. The analysis concentrated on three types of covariates related with violence: a) background/household (HH) characteristics; b) women’s characteristics and c) husband’s characteristics.

Background characteristics include: place of residence (urban/rural), province (province 1-7) ecological region: Mountain (northern mountain region of Nepal bordering China), Hill (mid hilly region) and Terai (plain region of Nepal), and wealth quintile of households. The wealth quintile in NDHS was calculated using principal component analysis based on household’s ownership of selected assets, e.g. televisions and bicycles; materials used for housing construction; and types of water access and sanitation facilities. Using these proxy indicators, the households were classified into five quintiles: the poorest, poorer, middle, richer, and the richest. [22]. The quintiles were later grouped into three categories for the current analysis: poor (poorest and poorer), middle and rich (richer and richest) as has been suggested by Chakraborty et al. [23]. Women’s characteristics include: age group of the women (15–19/20–24), caste of the women: Hill-Brahmin/Chettri (relatively advantaged), Terai caste (relatively advantaged living in southern plain region), Janajatis (indigenous groups) and Dalit/Others(disadvantaged populations), levels of education (no education/ primary/some secondary/SLC and above), current working status of women based on involvement in any work aside from own house work (yes/no), ever used contraception (yes/no), ideal family size as per her wish (0–2/3+) and decision-making status (yes/no).The definition of decision making status is based on the definition given on sustainable development goals 2016–2030, i.e. the SDG Indicator 5.6.1: ‘Proportion of women aged 15-49 years who make their own informed decisions regarding sexual relations, contraceptive use and reproductive health care’. Media exposure (frequency of reading newspaper or magazine, frequency of listening radio or frequency of watching television) were categorized into “Not at all”, less than once a week and at least once a week. Husband related covariates included levels of education (no education/ primary/some secondary/SLC and above), occupation (not working/ agricultural/non-agricultural/manual labor) and alcohol consumption (yes/no).

## Statistical analysis

Chi-square test was used to assess the association of sexual violence and other covariates with unintended pregnancy. Certain sub categories within categorical variables such as province (province 6 and 7); ecological region (mountain and hilly); husband education (None and primary) were merged due to few observations. Due to less number of cases in ‘not working’ category within ‘husband’s working status’ was not shown in the tables of bivariate and multivariate analysis. Multi-collinearity of the independent variable was checked before running multivariate models.

Multivariate logistic regression analysis was used to derive adjusted effects of sexual violence on unintended pregnancy. Initially, ever experience of sexual violence and covariates related to women’s fertility intention and decision-making (ever used contraception, ideal family size and her decision-making status), were included in the first model. Variables that showed significance in the first model (*p* < 0.05) were included in the second model which consisted socio-demographic characteristics of the women and their husbands. Odds ratios and 95% confidence intervals were presented in the results. Domestic violence weights from NDHS 2016 were applied during the analyses. We used the Svyset command to account for complex survey design and to provide unbiased estimates for odds ratio and their confidence intervals. The analyses were done using STATA version 15.0.

### Ethical considerations

NDHS 2016 survey protocol was reviewed and approved by Nepal Health Research Council and the institutional review board of ICF Macro International. We used de-identified publicly available dataset from DHS website (www.dhsprogram.com) for this analysis. Therefore, no separate ethical approval was required for this analysis. In NDHS 2016, the interviewers pursued informed consent from the women before interviews. Privacy and confidentiality was ensured during interviews by trained female interviewers who received special training to administer the domestic violence module. Permission was obtained from Measure DHS to use the data for further analysis.

## Results

### Prevalence of sexual violence and unintended last pregnancy

Table 1 presents the prevalence of sexual violence and unintended pregnancy among women who had childbirth experience in the last 5 years of the survey. Nearly one in 10 (9%) respondents reported to have ever experienced sexual violence from their husband. Nearly a quarter (23%) of the respondents reported unintended pregnancy.

**Table 1:** Percentage of respondents ever married age 15-24given birth in last 5 years who ever reported sexual violence by their current husband and unintended pregnancy

Characteristics	*n* = 560	% (95% CI)
Sexual violence	51	9.0 (6.3–12.8)
Physically forced her to have sexual intercourse with him when she did not want to	49	8.6 (6.0–12.5)
Physically forced her to perform any other sexual acts she did not want to	17	3.1 (1.78–5.2)
Forced her with threats or in any other way to perform sexual acts she did not want to	27	4.9 (3.0–7.8)
Unintended pregnancy (last birth)	127	22.7 (18.8–27.1)

### Background characteristics of respondents

Almost equal proportion of the respondents resided in the rural (50%) and urban (50%) areas. Most of the respondents were from province 2 (26%) followed by province 5 (19%) and province 1 (15%). More than half of the respondents (52%) were from Terai, 42% were from Hill and 6% from Mountain. Nearly half (48%) belonged to middle income households followed by poor (42%). Caste-wise, more than one third (34%) of the respondents were Janjatis (indigenous group) followed by Hill Brahmin/Chettri (25%). Most of the respondents were in the age group 20–24 (82%). Less than one fifth of the respondents had no education while almost half of them had either some secondary, school leaving certificate (SLC) or higher education. Nearly half (46%) of respondents were currently working. Nearly two fifth (38%) ever used contraception. Majority of respondents mentioned ideal family size to be less than 3. Just over a quarter (28%) of the respondents had media exposure at least once in a week while just over half (53%) of them had media exposure less than once in a week. Nearly 70% of husbands had some secondary or SLC and higher education. Most of the husbands were engaged in manual labor and agriculture-related employment. About two fifth (41%) of the respondents reported their husbands to be drinking alcohol (Table 2).

**Table 2:** Association between selected characteristics and unintended pregnancy (last birth)

Independent Characteristics	Total, n (%)	Unintended last births (%)	p-value
Total	560	22.7	
Geographical/Household characteristics			
Place of residence			
Urban	279 (49.8)	22.7	
Rural	281 (50.2)	22.7	0.981
Ecoregion			
Mountain/Hill	268 (47.9)	23.0	
Terai	291 (52.1)	22.4	0.892
Province			
Province1	85 (15.2)	17.1	
Province2	148 (26.4)	27.0	
Province3	82 (14.7)	36.3	
Province4	55 (9.7)	19.4	
Province5	107 (19.1)	14.4	
Province6 and 7	83 (14.9)	20.2	0.014*
Wealth index			
Poor	235 (41.9)	22.0	
Middle	267 (47.7)	23.6	
Rich	58 (10.4)	21.5	0.928
Caste			
Brahmin/Chettri-Hill	137 (24.6)	24.2	
Terai caste	112 (20.1)	25.2	
Janajatis	192 (34.3)	19.9	
Dalit/Others	118 (21.1)	23.2	0.785
Women’s Characteristics			
Age group			
15–19	103 (18.4)	29.7	
20–24	457 (81.6)	21.1	0.129
Education			
No education	98 (17.6)	15.4	
Primary	128 (22.9)	25.2	
Some secondary	200 (35.7)	24.1	
SLC and above	133 (23.8)	23.5	0.44
Working status			
No	301 (53.7)	22.7	
Yes	259 (46.3)	22.8	0.982
Ever used contraception			
No	345 (61.7)	22.5	
Yes	214 (38.3)	23.1	0.893
Ideal family size^a^			
0–2	487 (87.5)	24.8	
3+	70 (12.5)	8.7	0.014*
Decision making status^b^			
No	294 (58.6)	19.4	
Yes	208 (41.4)	26.4	0.118
Media Exposure^c^			
Not at all	106 (18.9)	25.7	
At least once a week	155 (27.7)	26.6	
Less than once a week	299 (53.4)	19.6	0.353
Husband’s characteristics			
Education^d^			
No education/Primary	170 (30.8)	20.7	
Some secondary	280 (50.7)	24.7	
SLC and above	102 (18.5)	20.4	0.633
Working Status^e^			
Did not work	15 (2.7)	7.7	
Agricultural	82 (14.8)	25.3	
Non-agricultural	77 (13.9)	27.4	
Manuallabor	379 (68.6)	21.7	0.501
Alcohol consumption			
No	333 (59.5)	20.9	
Yes	227 (40.4)	25.3	0.304
Sexual violence			
No	509 (91.0)	21.2	
Yes	51 (9.0)	38.1	0.010*

^a^Three non-numeric responses were excluded from the analysis

^b^58 cases who did not responses any of the decision making questions were not shown in the table

^c^Media exposure includes the exposure of newspaper, radio and television

^d^Eight don’t know cases were excluded from the analysis

^e^Seven missing cases were excluded from the analysis

*p<0.05

### Association between the independent characteristics and unintended pregnancy (last births)

Table 2 shows that highest proportion of unintended last births were reported in province 3 (36%), followed by province 2 (27%) and province 6 and 7 (20%). Proportion of unintended last births were higher among women who mentioned their ideal family size to be less than 3 (25%) compared to those reporting it to be more than or equal to 3 (9%). Nearly two fifth (38%) of women who had experienced sexual violence from their husbands had experienced unintended last births. We found no difference in proportion of unintended last births by other independent characteristics.

### Effect of sexual violence on unintended pregnancy (live births)

We created two multivariate logistic regression models to investigate adjusted effect of sexual violence on unintended pregnancy**.** In the first model, we included sexual violence, ever use of contraceptives, decision making status and perceived ideal family size. Variables that showed significance (*p* < 0.05) in the first model were included in the second model. In the second model (final model), variables selected from the first model and other socio-demographic covariates were included (Appendix).

Final model showed that women who experienced sexual violence were at 2.3 times higher risk to report an unintended birth (OR = 2.3; 95% CI = 1.1–4.8) compared to those who did not experience sexual violence. Women reporting ideal family size to be more than equal to three were 80% less likely to have unintended births’ than < 3 family size (OR = 0.2; 95% CI = 0.1–0.7). Women residing in province 3 were more likely to have unintended births than province 1 (OR = 3.2; 95% CI = 1.3–8.1). Women with primary (OR = 2.2; 95% CI = 1.1–4.4) and some secondary education (OR = 2.3; 95% CI = 1.0–5.2) were more likely to report unintended birth than women with no education (Table 3).

**Table 3:** Multivariate logistic regression analysis showing effect of sexual violence on unintended pregnancy in Nepal 2016

Characteristics	Model 1	Model 2
	OR (95% CI)	OR (95% CI)
Ever experienced Sexual violence		
No (Ref.)	1	1
Yes	2.9** (1.4–6.3)	2.3* (1.1–4.8)
Ideal family size		
0–2 (Ref.)	1	1
3+	0.3* (0.1–0.8)	0.2* (0.1–0.7)
Province		
Province1(Ref.)		1
Province2		2.3 (0.8–6.2)
Province3		3.2* (1.3–8.1)
Province 4		1.1 (0.4–2.9)
Province5		0.7 (0.3–1.8)
Province 6 and 7		1.1 (0.5–2.3)
Women’s education		
No education(Ref.)		1
Primary		2.2* (1.1–4.4)
Some secondary		2.3* (1.0–5.2)
SLC and above		2.4 (0.9–6.5)

only significant variables from model 1 and 2 shown in the table

*Ref.* Reference

**p < 0.01, *p < 0.05

## Discussion

This paper investigated the association of sexual violence with unintended pregnancy among Nepalese young women (15–24 years) who have had at least one child birth in the last 5 years of the NDHS survey. Nearly a quarter of women (22.7%) who delivered in last 5 years mentioned to have experienced unintended pregnancy. The proportion of unintended pregnancy among young women (15–24 years) who gave birth in last 5 years was slightly lower than previous estimates for Nepalese women of reproductive age (15–49 years) (24.6%) [14]. The differences in proportion of unintended pregnancy could be partly due to differences in age groups between two studies. Underestimation of burden of unintended pregnancy among young women in the current analysis is likely because we only included married women who had a live birth in last 5 years.

We found a strong association between ever experience of sexual violence from husbands and unintended pregnancy, aOR 2.3 (95% CI 1.1–4.8) among women who gave childbirth in the last 5 years which was consistent with findings from previous studies [6, 24–27]. The strength of association found in our study is similar to a study from Burundi [25], but greater than reported in studies from Colombia [27] and Peru [24]. These differences in strength of association could be due to variations in statistical models and confounder adjustment. Current findings indicate that women experiencing sexual violence in Nepal have significantly higher chances of unintended pregnancy irrespective of their education, household-wealth, and other important socio-demographic factors. Analysis of DHS data showed that partner violence was associated with increased risk of unintended pregnancy in 8 out of 10 countries [6].

Current analysis revealed that nearly 1 in 10 women (9%) had experienced sexual violence from their husbands ever in their life. The occurrence of sexual violence (9%) from husbands/spouses over the lifetime in our sample is slightly higher than reported among Colombian youths aged 13–24 years of age (6%) [27], and 20–24 years from Moldova (3%) (6)but is lower than among youths aged 20–24 years from other low and middle income countries: Bolivia(14%), Haiti(11%), Kenya(15%), Malawi(13%) and Bangladesh (26%) [6].

Unintended pregnancy among women who experienced sexual violence can occur through various pathways. First, male dominance in sexual decision making limits women’s control over fertility, access to and use of contraceptives [27–29]. Second, unintended pregnancy can occur due to refusal to use condom by men; and women’s fear of condom negotiation [29]. Third, women who experienced intimate partner violence may be more likely to experience pregnancy coercion or contraceptive sabotage increasing their risk of unintended pregnancy [30]. Fourth, chances of contraceptive failure [31], contraceptive discontinuation [32] and use of less reliable traditional methods [33] was reported to be higher among women experiencing intimate partner violence compared to those who did not experience violence.

We found that women who had primary and some secondary level education were more likely to experience unintended pregnancy than women who had no formal education. Women with above secondary level education showed the association in same direction but did not reach statistical significance. Current finding showing increased risk of unintended pregnancy among women with primary and some secondary education compared to women with no education is contradictory to earlier research among currently pregnant women from Nepal, which found no association between education and risk of unintended pregnancy [15]. The authors used a dichotomous variable (no education/illiterate and literate), while we used a variable with 4 levels of education. Differences in sample characteristics (age and pregnancy status) might explain this result or it might indicate a mediating role of sexual violence on the association of educational status with unintended pregnancy. Previous studies from Colombia and Bangladesh also found insignificant positive association between education and risk of unintended pregnancy [34]. Educated women may be more likely to reject traditional gender norms and may try to retaliate to their husband’s sexual violence which may have led to further revenge from husbands in a male dominated society [10]. A study from Burundi showed that frequency of partner violence was more common among women with primary, secondary and tertiary education than among women with no education, probably due to reporting bias [25]. Educated women may be more likely to report any kind of sexual violence and unintended pregnancy than women with less/no education [6]. Whereas, non-educated women might normalize sexual violence in marital relationship than their educated counterparts, which might have led to under-reporting of sexual violence and unintended pregnancy [34]. Despite these findings, importance of women’s education for positive health outcomes remains undisputed.

Although husband’s educational level was not associated with unintended pregnancy, husbands’ relative education to women may be another factor that played a role in sexual violence [10] and unintended pregnancy that needs further examination. Additionally, higher use of less effective traditional FP methods by educated women [35], might have led to higher rates of unintended pregnancy.

Women who expressed smaller ideal family size (less than 3 children), were more likely to experience unintended childbirth compared to women who wanted larger family size(more than 3). Adhikari et al. revealed similar findings from further analysis of NDHS 2011 data among pregnant women of 15–49 years from Nepal [15]. Since majority of Nepalese live in rural areas, women from rural areas might perceive greater benefit of having more children [15]. Further, women who wanted fewer children are more likely to be higher educated than women wanting more children [35]. Moreover, reproductive coercion and contraceptive sabotage by husbands might be more prevalent among women wanting fewer children than women wanting three or more children until desired sex composition is achieved since son preference was found to be equally prevalent among younger generations in Nepal [36].

Historically, sexual violence within marital relationship has remained an ‘open secret’ in Nepal [18]. Women who were illiterate, had lower autonomy to make decisions and who were raised in a family where violence was common were more likely to experience intimate partner violence [18]. Furthermore, lower autonomy and less inter-spousal communication were strongly associated with sexual violence among married young Nepalese women [10]. Social and cultural norms regarding women’s behavior requires them to be submissive and loyal to their husbands. Given the association between sexual violence and unintended pregnancy, strong implementation of Domestic Violence (Offense and Punishment) Act 2009 [37], and efforts to improve inter-spousal communication, and women’s autonomy needs to be in place to reduce the risk of unintended pregnancy in Nepal [10]. Research on dynamics of abuse and sexual violence in marital relationship is necessary to design prevention programs [38].

The current findings underscore the importance of multidimensional reproductive and sexual health services to married young women in Nepal for reducing unintended pregnancy and its consequences. Routine screening for sexual violence, and counseling or referral to appropriate services needs to be made available to all women. The family planning clinics and/or antenatal care sites can be used to provide information, counseling and services to reduce sexual violence and reproductive coercion [39]. Therefore, provision of screening and treatment for spousal violence within reproductive health care setting needs to be ensured [24]. Some promising violence prevention interventions (combining microfinance and gender equality training, improving couple communication and relationship skills, among others) need to be evaluated in Nepalese context for larger scale up [40].

Longer term reversible contraceptives reduce the risk of unintended pregnancy [41]. These may be more appropriate FP methods to women who are experiencing sexual violence but cannot live separately from abusive husbands. Similarly, female condoms and progesterone vaginal ring(PVR) can also enhance women’s control over their fertility [42]. However, availability and use of female condoms and PVR is very limited in Nepal [35] and needs to be promoted in future.

We used data obtained from a nationally representative survey. We analyzed a sample of women of young adult age (15–24 years) because evidence suggests that young women are more likely to experience violence. The findings presented here need to be interpreted in the light of some limitations. It was difficult to determine temporal relationship between sexual violence and unintended pregnancy due to cross-sectional nature of the data used for analysis as some studies have demonstrated that unintended pregnancy/induced abortion can also lead to violence [3, 43]. Additionally, there is a high chance of under-reporting of sexual violence because of its sensitive nature. Further, unintended births may have been under-reported after a child is born because of the joy associated with having a child. Additionally, women who terminated their unintended pregnancy through abortion or who experienced still birth or child death were not included in the sample which could have influenced our strength of association between unintended pregnancy and sexual violence.

## Conclusion

Sexual violence was strongly associated with unintended pregnancy among young women (15–24 years) who experienced child birth in last 5 years of the survey independent of ever use of contraceptives, women’s education, and other important socio-demographic factors. Therefore, assessment for sexual violence in marital relationship and referral to appropriate services can help to identify women at risk of unintended pregnancy. Hence, along with the provision of range of contraceptives, RH service sites need to be strengthened to provide assessment, counseling or referral services for sexual violence to prevent unintended pregnancies.

## Appendix

**Table 4:** Multivariate logistic regression analysis showing effect of sexual violence on unintended pregnancy in Nepal 2016

Characteristics	Model 1	Model 2
	OR (95% CI)	OR (95% CI)
Ever experienced Sexual violence		
No (Ref.)	1	1
Yes	2.9** (1.4 - 6.3)	2.3* (1.1 - 4.8)
Ever used contraception		
No (Ref.)	1	Not in the model
Yes	0.9 (0.6 - 1.5)	
Decision making status		
No(Ref.)	1	Not in the model
Yes	1.4 (0.8 - 2.3)	
Ideal family size		
0-2 (Ref.)	1	1
3+	0.3* (0.1 - 0.8)	0.2* (0.1 - 0.7)
Place of residence		
Urban(Ref.)		1
Rural		1.0 (0.6 - 1.6)
Ecological region		
Mountain/Hill(Ref.)		1
Terai		1.2 (0.6 - 2.4)
Province		
Province1(Ref.)		1
Province2		2.3 (0.8 - 6.2)
Province3		3.2* (1.3 - 8.1)
Province 4		1.1 (0.4 - 2.9)
Province5		0.7 (0.3 - 1.8)
Province 6 and 7		1.1 (0.5 - 2.3)
Wealth index		
Poor(Ref.)		1
Middle		0.9 (0.5 - 1.6)
Rich		0.7 (0.2 - 2.0)
Caste		
Brahmin/Chettri-Hill(Ref.)		1
Terai caste		0.9 (0.4 - 2.5)
Janajatis		0.5 (0.2 - 1.1)
Dalit/Others		1.0 (0.5 - 2.1)
Women’s age group		
15-19(Ref.)		1
20-24		0.8 (0.4- 1.4)
Women’s education		
No education(Ref.)		1
Primary		2.2* (1.1 - 4.4)
Some secondary		2.3* (1.0 - 5.2)
SLC and above		2.4 (0.9 - 6.5)
Women’s working status		
No(Ref.)		1
Yes		1.1 (0.6 - 1.9)
Media Exposure		
Not at all(Ref.)		1
At least once a week		1.1 (0.5 - 2.3)
Less than once a week		0.7 (0.3 - 1.4)
Husband’s education		
No education(Ref.)		1
Primary		1.4 (0.5-4.4)
Some secondary		1.5 (0.5 - 5.1)
SLC and above		1.3 (0.3 - 5.1)
Husband’s working Status		
Did not work		1
Agricultural		4.3 (0.5-36.5)
Non-agricultural		3.7 (0.5- 28.3)
Manual labor		3.5 (0.5 – 25.9)
Alcohol consumption by husband		
No(Ref.)		1
Yes		1.3 (0.8 - 2.3)

*Ref*. Reference

** *p*<0.01, * *p*<0.05

## Abbreviations

aOR: Adjusted odds ratio
CI: Confidence interval
DHS: Demographic and Health Survey
FP: Family planning
HH: Household
NDHS: Nepal Demographic and Health Survey
OR: Odds ratio
*p*-value: Probability value
PVR: Progesterone vaginal ring
SDG: Sustainable development goal
SLC: School leaving certificate
WHO: World Health Organization
